# Supplementary material for: A survey on common criteria (CC) evaluating schemes for security assessment of IT products
Source: PeerJ Comput Sci. 2021 Oct 26;7:e701. doi: 10.7717/peerj-cs.701 (PMC8576545; doi:10.7717/peerj-cs.701)
Supplement: Supplemental Information 1 [file peerj-cs-07-701-s001.pdf]

ACRONYMS

Common Evaluation Methodology	CEM
Denial of Service	DoS
Advanced Persistent Threats	APTs
Evaluation Assurance Levels	EALs
Security Functional Requirements	SFRs
Protection Profile	PP
Multi Functional Peripherals	MFPs
Security Objectives	SOs
Collaborative Protection Profile	cPP
Evaluation Technical Report	ETR
Target of Evaluation	TOE
Security Target	ST
Evaluation Consistency Review	ECR
Validation Report	VR
Validation Oversight Reviews	VORs
Check-In/Check-Out	CICO
Kick-Off	KO
Initial Validation Oversight Review	IVOR
Final Validation Oversight Review	FVOR
Test Validation Oversight Review	TVOR
Memorandum for the Record	MR
Impact Analysis Report	IAR
Evaluation Work Programme	EWP
Task Kick-off Meeting	TKM
Evaluation Progress Meeting	EPM
Assurance of ST Evaluation	ASE
Assurance of Vulnerability Analysis	AVA
Assurance for Testing	ATE
Single Evaluation Report	SER
Review Report	RR
Observation Report	OR
Certification Report	CR
Assurance Continuity	AC
Request for Interpretation	RI
Industrial Enabling Services	IES
Common Criteria documentation International Interpretations	IICC
UK Interpretations	UKI
Certification Work Programme	CWP
Certification Application Form	CAF
