# Supplementary material for: A survey on common criteria (CC) evaluating schemes for security assessment of IT products
Source: PeerJ Comput Sci. 2021 Oct 26;7:e701. doi: 10.7717/peerj-cs.701 (PMC8576545; doi:10.7717/peerj-cs.701)
Supplement: Supplemental Information 2 [file peerj-cs-07-701-s002.pdf]

ACRONYMS

Information Technology	IT
Common Criteria	CC
Information Systems	IS
United States	US
United Kingdom	UK
Universal Serial Bus	USB
Common Criteria Protection Profile	CCPP
Internet of Things	IoT
Certification Body	CB
Global Cybersecurity Index	GCI
Common Criteria Recognition Arrangement	CCRA
Commercial off-the-shelf	COTS
Common Criteria Testing Laboratory	CCTL
Product Compliant List	PCL
Technical Committee	TC
Common Criteria Evaluation and Validation Scheme	CCEVS
National Information Assurance Partnership	NIAP
National Security Agency	NSA
National Institute of Standards and Technology	NIST
National Voluntary Lab Accreditation Program	NVLAP
Technical Rapid Response Team	TRRT
Singapore Common Criteria Scheme	SCCS
Cyber Security Agency of Singapore	CSA
Singapore Accreditation Council	SAC
International Organization for Standardization	ISO
Certified Product List	CPL
National Scheme Communication	NSC
Historical Product List	HPL
Netherlands Scheme for Certification in the Area of IT Security	NSCIB
Information Technology Security Evaluation Facility	ITSEF
Commercial Evaluation Facilities	CLEFs
Scheme Information Notices	SINs
CESG Test Laboratory General Operational Requirements	CGOR
Point of Contact	POC
CLEF Progress Report	CPR
Information and Communication Technology	ICT
Management Committee	MC
Executive Subcommittee	ES
Assurance Continuity Maintenance Report	ACMR
